# From particle count to residence time: a kinetic framework for atherogenic risk

**DOI:** 10.3389/fcvm.2026.1898635

**Published:** 2026-09-03

**Authors:** Esteban Larronde

**Affiliations:** 1 Diplomatura en Medicina del Estilo de Vida, Physician, Chos Malal, Universidad Nacional del Comahue, Neuquén, Argentina; 2 Diplomatura en Medicina del Estilo de Vida, Facultad de Ciencias Médicas, Universidad Nacional del Comahue

**Keywords:** apolipoprotein B, electronegative LDL, fractional catabolic rate, glycocalyx, PCSK9, residence time

## Abstract

Two sequential advances have narrowed the gap between lipid measurement and atherogenic mechanism. Mendelian randomization established that coronary risk tracks apolipoprotein B (ApoB) particle count, not cholesterol content: when natural discordance exists between low-density lipoprotein cholesterol (LDL-C) and ApoB, risk follows ApoB reduction independently of LDL-C. The 2026 ACC/AHA dyslipidemia guideline formalized this advance, placing ApoB above LDL-C as a risk measure in discordance contexts. That particle-count model fails systematically before a set of clinical paradoxes sharing a common feature: identical ApoB concentrations generating radically different cardiovascular risk depending on metabolic and endothelial context. Hemodialysis patients carry ten- to twenty-fold excess cardiovascular risk with normal ApoB, and statins do not reduce events in dialysis despite reducing LDL-C. PCSK9 loss-of-function (LOF) variants produce an 88% coronary risk reduction at LDL-C of 100 mg/dL, a magnitude approximately three times what the logarithmic dose-response predicts. Patients with systemic lupus erythematosus and rheumatoid arthritis develop accelerated atherosclerosis with normal lipids. Male endurance athletes with favorable lipid profiles show coronary artery calcium scores equivalent to sedentary controls with traditional risk factors. I propose that atherogenic risk is a function of the plasma residence time (RT) of ApoB-containing particles, determined by two separable kinetic components: FCR_est (structural, genotype-dependent fractional catabolic rate, d-1) and FCR_cin (functional, kmod-dependent clearance efficiency, dimensionless 0−1), modulated by an endothelial permeability function proxied by the urinary albumin-to-creatinine ratio (UACR). The framework is expressed as: Atherogenic Load = [ApoB]×(1/FCR)×kmod×f(UACR,glycocalyx). The model generates a four-group functional classification with qualitatively distinct therapeutic implications: the rate-limiting step is the LDLR receptor in Group 1a [familial hypercholesterolemia, PCSK9 gain-of-function (GOF) variants], the ApoB-100 ligand in Group 2 (metabolic syndrome, uremia, autoimmunity), and the endothelial gate in Group 3. Treating all groups with the same LDL-C reduction strategy is a category error with quantifiable consequences. The five core predictions of the framework are falsifiable with currently available stable-isotope kinetic and anion-exchange chromatography technology.

## 1 Introduction

Preventive cardiology has operated under three successive paradigms, each a genuine advance over its predecessor, each insufficient before a distinct class of clinical situations the model cannot resolve.

For four decades, LDL-C was the primar*y* axis of cardiovascular risk stratification. The Heart Protection Study and the Cholesterol Treatment Trialists meta-analysis consolidated its position (1, 2). The model treats risk as a function of plasma cholesterol stock, ignoring how many particles carry that cholesterol, how long they circulate, in what oxidative conditions they do so, and whether the endothelium they must cross is intact. These four omissions generate four distinct classes of prediction error that accumulate into irresolvable paradoxes.

Ference et al. established, first in a 2012 Mendelian randomization (MR) analysis (3) and then in a 2017 integrative study (4), establishing that cardiovascular risk is proportional to ApoB particle concentration integrated over the patient's lifetime. The 2026 ACC/AHA guideline elevated ApoB to a superior risk measure compared to LDL-C, particularly in discordance contexts (5). Morze et al. confirmed in 207,368 UK Biobank participants that ischemic heart disease risk follows total ApoB particle count independently of particle type or size, with Lp(a) adding significant independent risk beyond ApoB (6).

The cumulative-exposure model treats the particle as a passive projectile whose only relevant variable is concentration. It does not consider the state of the particle at each moment of its circulating time, nor the conditions of the endothelium it must cross. The natural experiments described in Section 3 are situations where equal particle counts generate radically different risk, depending on oxidative environment and endothelial integrity.

**Figure 1:**
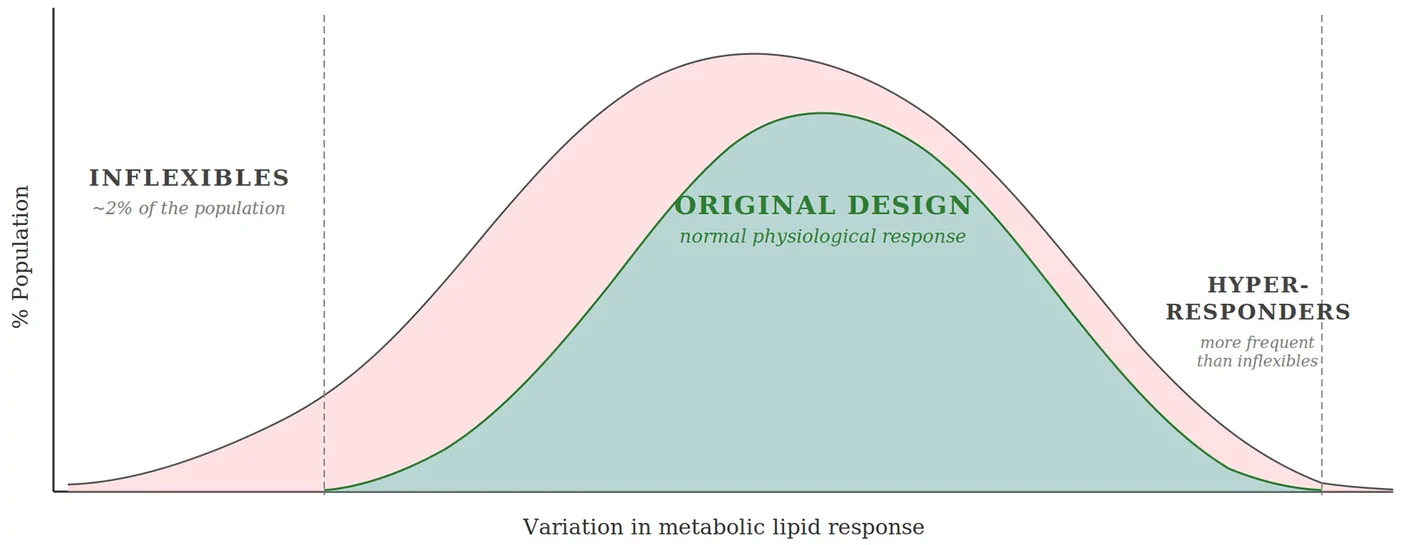
Hypothetical distribution of biological variability in population lipid metabolic response. The filled inner curve represents the majority group with normal physiological response (original design); the outer curve illustrates the full spectrum of variation. The asymmetry is intentional: the left extreme (inflexibles) groups genetically rigid forms of low prevalence, FH, PCSK9 GOF, monogenic obesity, lipodystrophy; the right extreme (hyperresponders) is more frequent though equally a minority. A guideline that assigns the same numerical LDL-C target to all three profiles operates on this distribution as if it were symmetric. Elaborated by Larronde, 2025.

The biological rationale for adding a kinetic dimension is grounded in population-level variability of lipid metabolic response. At the left extreme of that distribution are patients whose lipid system cannot adjust regardless of environmental input: familial hypercholesterolemia (FH), PCSK9 GOF, monogenic obesity, lipodystrophy. At the right extreme are hyperresponders whose system reacts disproportionately to minor stimuli. The majority occupy a flexible center for whom lifestyle intervention is the primary lever. A guideline that assigns the same numerical LDL-C target to all three profiles operates on this distribution as if it were symmetric (Figure 1).

### 1.1 Unresolved clinical and mechanistic gaps: the motivation for a kinetic framework

Four classes of prediction failure motivate the step from particle count to kinetics:

(1) Equal ApoB, unequal risk by metabolic context. Hemodialysis patients have ApoB concentrations indistinguishable from matched controls, yet carry ten- to twenty-fold excess cardiovascular mortality. Statins reduce LDL-C in this population without reducing events (4D, AURORA trials).3,4 The ApoB-years model predicts proportional benefit from ApoB reduction regardless of metabolic context; it cannot explain why the same reduction fails when the chemical environment of the particle is altered by uremic toxins.
(2) Equal LDL-C reduction, unequal benefit by mechanism. CETP inhibitors that reduce LDL-C by emptying cholesterol from the particle without withdrawing it from circulation produce no cardiovascular benefit (evacetrapib: ACCELERATE; dalcetrapib: dal-OUTCOMES).5 Anacetrapib, which reduces LDL-C by increasing ApoB clearance (FCR +23% by stable isotopes; Millar et al. (7), produces benefit (8). The difference is not captured by LDL-C or ApoB concentration. It is captured by RT.
(3) Disproportionate protection from genetic variants that accelerate clearance. PCSK9 LOF variants produce 88% coronary risk reduction at LDL-C of 100 mg/dL, approximately three times the protection predicted by the logarithmic LDL-C dose-response (9). Cariou et al. documented FCR increased by 256% in carriers of a dominant-negative PCSK9 variant, producing RT of approximately 1.4 days, within the latency phase of the oxidative modification sequence before significant ApoB conformational change (10).
(4) Atherogenesis driven by the metabolic environment, not the lipid level. Rheumatoid arthritis (RA) and systemic lupus erythematosus (SLE) produce cardiovascular risk 4- to 50-fold above matched controls with normal or low LDL-C and ApoB. Tumor necrosis factor (TNF) inhibition raises LDL-C in RA while reducing cardiovascular events, the opposite of what both prior models predict (11). The common feature is chronic inflammatory activation of oxidative modification pathways (kmod), independent of particle concentration.

Each gap maps to a specific term in the RT equation: gap (1) to kmod and FCR_cin; gap (2) to 1/FCR as the operative variable rather than cholesterol content; gap (3) to the dual mechanism of FCR_est x g(kmod) from PCSK9 LOF; gap (4) to kmod as the primary atherogenic driver in Group 2. The constructs FCR_est, FCR_cin, and kmod are introduced in Section 2 only after this mapping is established.

## 2 The kinetic framework

### 2.1 Apob as the unit of vehicle count

Each atherogenic particle contains exactly one ApoB-100 molecule, invariant and independent of cholesterol content (12, 13). ApoB concentration is the direct count of atherogenic vehicles in traffic. In discordance contexts [type 2 diabetes (T2DM), metabolic syndrome, hypertriglyceridemia] LDL-C underestimates particle count because cholesterol-depleted particles each still carry one ApoB. The MR evidence confirms that risk follows the number of vehicles, not the cargo they carry (4). The step this paper proposes is to recognize that equal numbers of vehicles generate different risk depending on how long each has been in circulation and in what oxidative state it arrived at the endothelium.

A note on ApoB-48 and chylomicron remnants. The RT framework as developed here applies to ApoB-100-containing lipoproteins [VLDL, IDL, LDL, and Lp(a)], which are cleared via LDLR-mediated endocytosis. The endothelial glycocalyx has a pore size of approximately 7 nm and effectively excludes particles above approximately 10 nm under intact conditions. Cheng et al. demonstrated that healthy glycocalyx reduces uptake of 10 nm polyethylene glycol-coated gold nanoparticles to approximately 1% compared to degraded glycocalyx, a 6–7x difference attributed to the 7 nm pore structure (14). This was confirmed *in vivo* in mouse carotid arteries: intact glycocalyx showed minimal 10 nm nanoparticle infiltration; degraded glycocalyx showed 2.5-fold greater infiltration (15). Kang et al. provided direct structural characterization of glycocalyx-LDL interaction in rat aorta, confirming that glycocalyx modulates LDL access to the subendothelial space in a structure-dependent manner (16).

Native chylomicrons are 100–1,000 nm, categorically excluded by an intact glycocalyx. Post-lipolysis remnants can be reduced to 30–80 nm, still 4- to 11-fold above the effective exclusion threshold. LDL itself (∼22 nm) exceeds the 7 nm pore and yet penetrates the intima physiologically via active caveolin-mediated transcytosis, not passive filtration, a regulated, saturable, low-rate process (17). Chylomicron remnants at 30–80 nm lack an equivalent dedicated transcytosis pathway, and their atherogenic mechanism depends heavily on glycocalyx degradation, placing them within the Group 3 f(UACR, glycocalyx) amplification mechanism. Extension of the RT model to ApoB-48 kinetics, incorporating LPL-dependent lipolysis as the primary FCR determinant, is deferred to future work.

### 2.2 Fractional catabolic rate, residence time, and the two-component model

The FCR expresses what fraction of the plasma ApoB pool is cleared per unit time (units: d-1); its inverse is the RT (units: days). In normolipidemic subjects, LDL-ApoB FCR is 0.31–0.37 d^-1^ (RT approximately 2.5 days) (56, 57). In heterozygous FH it falls to 0.16–0.24 d^-1^ (RT approximately 4.5 days); in homozygous FH it exceeds 0.17 d-1 (RT exceeding 6 days) (15).

FCR has two mechanistically separable components with distinct therapeutic consequences:

FCR_est (structural, units: d-1): determined by functional LDLR receptor density. Genotype-fixed, reduced in Group 1a by a structural defect operating independently of plasma metabolic state. High-efficacy pharmacology targeting LDLR is the only first-order intervention.
FCR_cin (functional, dimensionless 0–1): the efficiency of receptor-ligand recognition, expressed as g(kmod), where kmod is the net rate constant of oxidative ApoB modification (d-1). FCR_cin is reduced in Group 2 because kmod degrades the ApoB-100 ligand, generating a kinetic dead zone: the modified particle is no longer recognized efficiently by LDLR or scavenger receptors, accumulating beyond its normal clearance window.

The combined relationship FCR(t) = FCR_est x g[kmod(t)] is a first-order multiplicative approximation. The product preserves units of d-1. This formulation may overestimate the kinetic defect in the zone of moderate oxidative modification, as FCR_cin decreases gradually without reaching zero under biological conditions.

For triglyceride-rich lipoproteins (VLDL, IDL, and chylomicron remnants), plasma RT is co-determined by the efficiency of endothelial lipolysis via lipoprotein lipase (LPL) anchored by glycosylphosphatidylinositol-anchored high-density lipoprotein-binding protein 1 (GPIHBP1), regulated by ApoC-III (inhibitor) and the ANGPTL3/4/8 system. LOF variants in APOC3 reduce TRL RT and coronary risk by 41% in 75,725 participants (Jorgensen et al. (18), providing direct MR evidence for a lipolytic FCR mechanism acting on atherogenic risk independently of LDL-C. ANGPTL3 LOF produces pan-lipoprotein reduction and cardiovascular protection through the same kinetic route (Graham et al. (19). Critically, ApoC-III expression rises in insulin resistance, in active inflammation, and in metabolic syndrome, the same conditions that elevate kmod and generate the Group 2 kinetic phenotype. These proteins are mediators through which the metabolic environment extends TRL RT, downstream of the same kmod state that also degrades the ApoB-100 ligand. A formal extension incorporating FCR_lip as a third FCR component (LPL-dependent, ApoC-III and ANGPTL-regulated) is identified as a priority for future work.

### 2.3 The oxidative modification sequence and L5 generation

Prolonged plasma exposure generates atherogenic modification through a temporal sequence. The proximal event is desialylation of ApoB-100 residues, the first step in the L1-to-L5 conversion cascade, documented by Tertov et al. as an *in vivo* process occurring in human plasma (20). Progressive oxidative modification follows, driven by a spectrum of kmod inputs: metabolic reactive oxygen species, carbamylation (uremia, smoking), acrolein adducts, and inflammatory cytokine-driven oxidation.

The *in vivo* correlation between RT and ApoB oxidative modification was documented directly by Pietzsch et al. in subjects with Familial Defective ApoB-100: HAVA content, a specific marker of Pro/Arg residue oxidation, in LDL2 correlated with RT measured by stable isotopes at r = 0.976 (*p* < 0.0001) in controls and r = 0.893 (*p* = 0.003) in carriers (21). Critical epistemic note: this study included *n* = 6 controls and *n* = 7 carriers; the r = 0.976 coefficient has a 95% CI extending down to approximately 0.76. This finding serves a generative epistemic function, it motivated and is consistent with the hypothesis, but is insufficient to establish it. Replication in adequately powered cohorts is required before any quantitative predictive application.

The terminal product of this sequence is the L5 subfraction, the most electronegative LDL fraction by anion-exchange chromatography, representing ApoB-100 with extensive oxidative and conformational modification. L5 binds LOX-1 on endothelial cells rather than LDLR, triggers nuclear factor kappa B (NF-kB)--mediated inflammatory signaling through LOX-1 (22), upregulates PCSK9 expression (23), and further reduces LDLR surface density. This creates a positive feedback loop: elevated RT generates L5, L5 activates LOX-1, LOX-1 upregulates PCSK9, PCSK9 degrades LDLR, reduced LDLR extends RT of subsequent particles. The overall L5 biology is reviewed in Rivas-Urbina et al. (24).

### 2.4 The unifying equation

AtherogenicLoad=[ApoB]×(1/FCR)×kmod×f(UACR,glycocalyx)

In this formulation, kmod operates at two biologically distinct sites: (i) on clearance efficiency via FCR(t) = FCR_est x g[kmod(t)], already captured in the 1/FCR term; and (ii) on the pro-retentive and pro-inflammatory phenotype of the particle once it reaches the subendothelium (L5-mediated LOX-1 activation and increased proteoglycan binding affinity). The explicit kmod term represents this second, downstream component and does not duplicate the effect already embedded in g(kmod).

**Figure 2:**
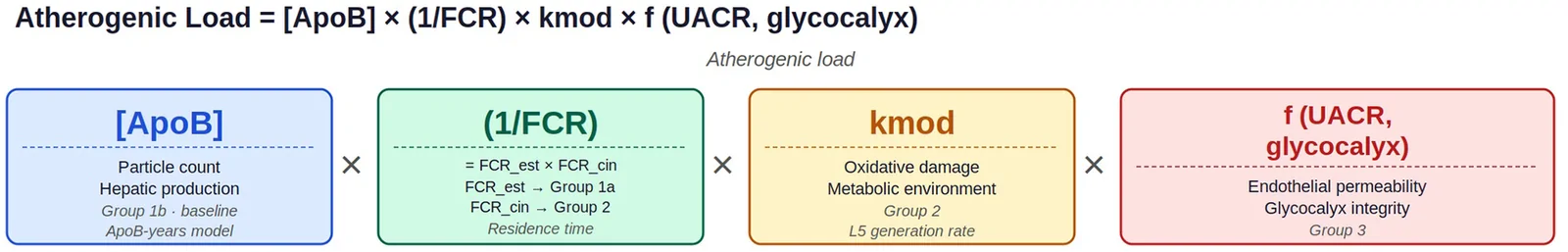
Unifying equation of the RT framework. Atherogenic Load=[ApoB] x (1/FCR) x kmod x f(UACR, glycocalyx). kmod: net rate constant of chemical LDL modification toward electronegativity (enzymatic, non-enzymatic, and oxidative pathways; units: d-1). FCR**(t)** = FCR_est x g[kmod**(t)**], where FCR_est is the structural clearance capacity (LDLR density/activity; units: d-1) and g(kmod) is a dimensionless factor (0-1) of receptor-ligand recognition efficiency (FCR_cin). kmod acts on clearance efficiency via g(kmod) and independently on particle retention toxicity via L5/LOX-1/proteoglycan binding: biologically separable mechanisms without double-counting. f(UACR, glycocalyx): endothelial permeability function proxied by UACR. Each term corresponds to a functional group: [ApoB] to Group 1b and baseline; (1/FCR) with FCR_est to Group 1a, with FCR_cin to Group 2; kmod to Group 2; f(UACR, glycocalyx) to Group 3. Elaborated by Larronde, 2025.

The f(UACR, glycocalyx) term proxies endothelial gate integrity. The glycocalyx acts as a size- and charge-selective barrier to subendothelial LDL access. Glycocalyx degradation by oxidized LDL and inflammatory mediators increases transcapillary LDL permeability (25, 26). Rabelink and de Zeeuw documented that glycocalyx disruption is the mechanistic link between albuminuria and vascular disease (27). Salmon and Satchell extended this to systemic microvascular permeability (28) UACR anchors the model to routinely measurable clinical data. The four terms of the equation and their correspondence to each functional group are illustrated in Figure 2.

## 3 Natural experiments where prior models fail

The following situations were not selected *post-hoc* to construct the model. They are pre-existing clinical paradoxes that the LDL-C and ApoB-years models have not resolved without *ad hoc* adjustments. I present them as the observations that motivated the framework. This means the RT model must be credited with *post-hoc* narrative consistency, not prospective prediction, a distinction addressed explicitly in the limitations section.

### 3.1 Hemodialysis: excess risk with normal apoB and statin failure

The 4D trial (atorvastatin vs. placebo in 1,255 dialysis patients with T2DM) and AURORA (rosuvastatin vs. placebo in 2,776 hemodialysis patients) demonstrated that statins reduce LDL-C by 30%–43% but do not reduce cardiovascular events in dialysis (29, 30). Cardiovascular mortality in this population runs ten- to twenty-fold above age-matched controls despite normal or low LDL-C. This double paradox, excess risk with normal lipids, and statin inefficacy despite lipid reduction, is the most direct challenge to both prior models.

The RT framework predicts this result through the kinetic dead zone mechanism. Uremia generates three distinct classes of ApoB-modifying agents: carbamylated LDL (Apostolov et al. (31, 32) and Wang et al. (33), acrolein-modified LDL (Kobayashi et al. (34) and Watanabe et al. (35), and a marked increase in the L5 subfraction documented independently by Chang et al. (36), Lobo et al. (37), Hirowatari et al. (38), and Chang et al. (39) Ikewaki et al. measured LDL-ApoB FCR directly using stable isotopes in hemodialysis patients and documented significantly delayed catabolism compared to controls (40). The mechanistic logic: kmod from uremic toxins degrades the ApoB-100 ligand, generating a kinetic dead zone where the modified particle is recognized neither efficiently by LDLR nor by hepatic scavenger receptors. Statins reduce production and modestly increase LDLR expression, acting on FCR_est, but cannot repair FCR_cin when the ligand is chemically modified. The statin ceiling in dialysis is determined by the kinetic state of the ligand, not by receptor availability.

### 3.2 CETP inhibition: divergent outcomes by mechanism

Evacetrapib (ACCELERATE, *n* = 12,092) raised HDL-C by 130% and reduced LDL-C by 37% with no cardiovascular benefit (41). Dalcetrapib produced modest HDL-C increase and no benefit. Anacetrapib (REVEAL, *n* = 30,449) reduced major coronary events by 9% relative risk (8).

The MR analysis of Ference et al. (4) established the first explanatory layer: reducing cholesterol content without reducing ApoB particle count produces no cardiovascular benefit. The RT framework adds a mechanistic second layer: Millar et al. showed using stable isotopes that anacetrapib reduces LDL by increasing ApoB clearance (FCR +23%), not by reducing cholesterol content per particle (7). Anacetrapib shortened RT. Evacetrapib and dalcetrapib emptied cholesterol from the particle without withdrawing it from circulation, leaving RT unchanged and leaving the L5 generation sequence uninterrupted.

### 3.3 PCSK9 loss-of-function: disproportionate protection

Cohen et al. reported that PCSK9 LOF variants reducing LDL-C by approximately 28% produce an 88% reduction in coronary heart disease risk over 15 years (9). This magnitude is approximately three times the risk reduction predicted by the logarithmic LDL-C dose-response, and remains unexplained by the ApoB-years model even after accounting for lifetime exposure.

The RT framework predicts this through two concurrent mechanisms: (1) Cariou et al. documented that carriers of a PCSK9 dominant-negative variant have LDL-ApoB FCR increased by 256%, producing RT of approximately 1.4 days (10). At this RT, particles circulate almost entirely within the latency phase of the modification sequence, generating minimal L5 and retaining near-native LDLR affinity. (2) PCSK9 upregulates LOX-1 expression via NF-kB and inflammatory cytokine signaling (23). LOF variants reduce this upregulation, decreasing the LOX-1-mediated positive feedback loop. The dual mechanism, shortened RT plus reduced LOX-1 amplification, explains protection exceeding what ApoB concentration reduction alone would predict.

### 3.4 Autoimmune atherogenesis: the lipid paradox of RA and SLE

Women with SLE have age-specific myocardial infarction rates 50-fold higher than age-matched Framingham controls in the 35–44 age group (42) RA presents a related paradox: patients develop accelerated subclinical atherosclerosis with LDL-C frequently below population means. Giles et al. documented an inverse association between LDL-C and subclinical coronary atherosclerosis in RA patients (43) TNF inhibition increases LDL-C in RA while reducing cardiovascular risk (11), consistent with suppression of an inflammatory kmod that was generating atherogenic L5 despite low measured cholesterol.

The RT framework predicts both paradoxes through chronic inflammatory kmod elevation. TNF-alpha and interleukin-6 (IL-6) accelerate LDL oxidative modification independent of LDL-C level. Chan et al. documented L5 levels 3.4-fold higher in SLE patients than in matched controls (44). Chang et al. reported an odds ratio of 4.94 for subclinical atherosclerosis associated with elevated L5 in RA patients (45). Risk follows kmod-driven L5 generation, not particle concentration.

### 3.5 Antioxidant trial failure

The HOPE trial (vitamin E), CARET (beta-carotene), and SELECT (vitamin E plus selenium) all failed to reduce cardiovascular events despite mechanistic rationale targeting LDL oxidative modification (46–48). The RT framework explains this through two mechanisms. First, kmod is episodic, not tonic. Postprandial oxidative stress, inflammatory flares, exercise-induced Nox2 activation, and acute infections generate discrete peaks of ApoB modification that stationary antioxidant coverage cannot neutralize. Second, at pharmacological doses, alpha-tocopherol inhibits glutathione S-transferase pi (48) and can attenuate the NRF2-mediated adaptive antioxidant response (47). Exercise-induced hormesis, which activates NRF2 and increases PON1 activity in HDL, is mechanistically distinct from supplemented coverage, explaining why regular physical activity reduces cardiovascular risk while vitamin E supplementation does not.

### 3.6 Male endurance athletes and coronary artery calcium

High-volume male endurance athletes (greater than 2,000 MET-min/week) have coronary artery calcium (CAC) scores equal to or higher than non-athletes with comparable traditional risk factors, despite lower LDL-C and lower ApoB. The sex specificity is consistent: female athletes of equivalent training volume do not show increased coronary calcification compared to sedentary controls.

The ApoB-years model predicts the opposite result: chronic aerobic training upregulates hepatic LDLR, accelerates LDL-ApoB FCR, and reduces ApoB production. The observed CAC elevation at equivalent or lower ApoB directly contradicts this prediction. The RT framework predicts the result through two mechanisms acting in parallel: (1) Episodic kmod elevation from Nox2 activation. During sustained exercise, the dominant reactive oxygen species generator is sarcolemmal NADPH oxidase (Nox2), not mitochondrial Complex I. High-volume sessions generate discrete oxidative peaks accelerating L1-to-L5 conversion, even when resting lipid profile is favorable. (2) Mechanical shear-mediated glycocalyx degradation at extreme training volumes increases transcapillary LDL permeability. Both mechanisms are sex-dimorphic: estradiol maintains glycocalyx structural integrity, has direct antioxidant effects on ApoB-100 preventing the misfolding that generates electronegative conformers (49), and reduces net kmod burden. This explains why female athletes are protected from the same training volume that elevates CAC in male counterparts.

## 4 Functional classification of atherogenic risk

The four groups below are regions in a continuous distribution of kinetic phenotypes, not rigid diagnostic categories. Their function is to identify the dominant determinant of prolonged RT, because that determinant, not the absolute ApoB level, dictates the first-order intervention. Patients may belong to more than one group simultaneously (Table 1).

**Table 1:** Functional classification: dominant mechanism, entities, and first-order intervention.

Group	Dominant mechanism	Representative entities	RT determinant	First-order intervention
1a	Structural: reduced FCR_est, LDLR defect, genotype-fixed	FH het/hom, PCSK9 GOF, FDB	FCR_est fixed low; RT structurally prolonged	High-efficacy LDLR pharmacology (statin + iPCSK9; apheresis in homozygotes)
1b	Structural: absent ApoB production, MTP defect	Abetalipoproteinemia, FHBL	Production near zero; RT variable irrelevant	Not applicable
2	Environmental: reduced FCR_cin, kmod elevated, kinetic dead zone	MetS, T2DM, CKD/uremia, autoimmunity, smoking, HIV	kmod degrades ligand; FCR_cin falls; RT prolonged despite intact LDLR	Metabolic correction first: weight loss, glycemic control, anti-inflammatory
3	Endothelial gate: glycocalyx degradation (second-level hypothesis)	Any Group 1a or 2 with microalbuminuria	Subendothelial retention extended beyond plasma RT prediction	Endothelial restoration: investigational

### 4.1 Group 1a: structural atherogenic RT

FCR_est is reduced by a structural defect operating independently of plasma metabolic state. Representative entities: heterozygous and homozygous FH, PCSK9 GOF variants, Familial Defective ApoB-100. The causal direction is genotype-fixed: reduced FCR_est leads to prolonged RT, driving time-dependent oxidative modification and L5 generation regardless of diet, exercise, or metabolic control. Tremblay et al. documented 50%–109% increased VLDL-ApoB production in FH patients carrying the same null LDLR mutation, driven by a constitutively active SREBP cleavage-activating protein/sterol regulatory element-binding protein 2 (SCAP/SREBP-2) loop from hepatic cholesterol deficit (50). This production amplification compounds the clearance defect. First-order intervention: high-efficacy LDLR pharmacology, high-intensity statins plus PCSK9 inhibitors (iPCSK9); LDL-apheresis in homozygotes.

### 4.2 Group 1b: structural protective RT

ApoB production is structurally reduced or absent through defect in VLDL assembly. Representative entities: abetalipoproteinemia (absent MTP), familial hypobetalipoproteinemia (FHBL). RT as a variable loses practical relevance because there are insufficient particles to generate atherogenic load. Group 1a fails at exit (clearance), Group 1b fails at entry (production), they predict opposite cardiovascular trajectories and must not be conflated.

### 4.3 Group 2: environmental or context-dependent RT

LDLR and ApoB-100 ligand function correctly under physiological conditions. FCR_cin is reduced because elevated kmod degrades the ApoB-100 ligand, creating a kinetic dead zone. Representative entities: metabolic syndrome, T2DM, visceral obesity, chronic kidney disease (CKD), tobacco smoking, prolonged Western dietary pattern, autoimmune diseases in active phase, chronic infection including HIV.

**Figure 3:**
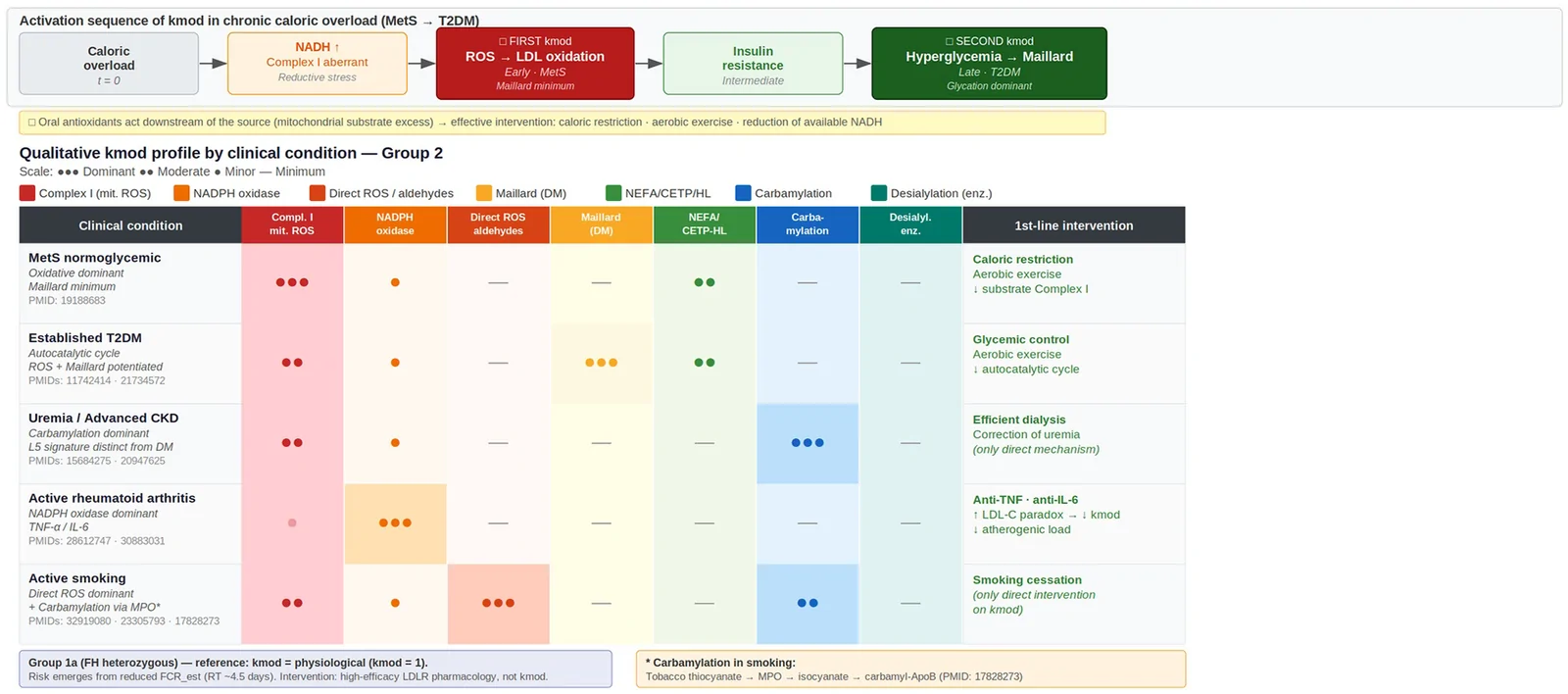
Activation sequence of kmod in chronic caloric overload. Reductive stress from NADH excess drives aberrant Complex I activity, generating the first kmod peak (ROS-mediated LDL oxidation) in early metabolic syndrome (MetS). Progression to insulin resistance and then hyperglycemia activates a second, mechanistically distinct kmod pathway (Maillard glycation) that becomes dominant in established type 2 diabetes mellitus (T2DM). The two kmod peaks generate distinct L5 subfraction signatures and explain why glycemic control, not antioxidant supplementation, is the effective first-order intervention in established T2DM. Oral antioxidants act downstream of the mitochondrial substrate excess; they cannot neutralize the upstream NADH-driven reductive stress. Elaborated by Larronde, 2025. Relative kmod pathway contribution across five Group 2 clinical conditions. Bar length indicates relative dominance of each pathway within that condition (full bar, dominant; half bar, moderate; narrow bar, minor; dash, negligible). MetS (metabolic syndrome): Complex I-driven oxidative stress dominant; Maillard contribution negligible; intervention targets upstream substrate excess via appetite/satiety regulation and aerobic exercise. T2DM (type 2 diabetes mellitus): second dominant kmod from Maillard glycation superimposed on residual oxidative stress; both pathways generate L5 but with distinct chemical signatures. CKD (chronic kidney disease) uremia: carbamylation-dominant L5 signature distinct from diabetes; only direct mechanism on kmod is uremia correction. RA (rheumatoid arthritis): NADPH oxidase dominant via TNF-alpha/IL-6; anti-cytokine therapy raises LDL-C paradoxically while reducing kmod and atherogenic load. Active smoking: direct ROS dominant plus myeloperoxidase (MPO)--mediated carbamylation (*tobacco thiocyanate via MPO to isocyanate to carbamyl-ApoB). Contributions are qualitative estimates based on mechanistic inference. Group 1a (FH) reference: kmod=physiological; risk in Group 1a arises from reduced FCR_est (RT approximately 4.5 days), not from elevated kmod. Elaborated by Larronde, 2025.

The hemodialysis paradox is the conceptual demonstration in its most extreme form: maximal kmod from uremic toxins generating maximal kinetic dead zone, explaining both the excess risk and the statin failure simultaneously. Direct kinetic evidence that metabolic correction increases FCR: weight loss in metabolic syndrome increased LDL-ApoB FCR by 27% (*p* < 0.05) without modifying production, measured with stable isotopes by Ng et al. (51). Mediterranean diet increased LDL-ApoB FCR by 30.4% (*p* = 0.02) independently of weight loss, measured by Richard et al. (52). Both findings document that the FCR_cin defect is reversible by correcting the metabolic environment, a prediction unique to the RT framework. The kmod activation sequence and the qualitative profile across five Group 2 clinical conditions, with their dominant pathways and first-order interventions, are shown in Figures 3 respectively. First-order intervention: metabolic correction before or alongside lipid pharmacology.

### 4.4 Group 3: endothelial gate degradation (second-level hypothesis)

This group carries the lowest epistemic confidence in the framework and is presented explicitly as a second-level hypothesis. The endothelial glycocalyx acts as a charge- and size-selective barrier to subendothelial LDL access. When oxidized LDL and inflammatory mediators degrade the glycocalyx, transcapillary LDL permeability increases and subendothelial retention time extends beyond what plasma RT and kmod alone would predict (25–28) Group 3 amplifies risk generated by Groups 1a or 2. UACR proxies glycocalyx integrity in clinical practice. Group 3 is not invoked as the primary explanatory mechanism in any of the paradoxes of Section 3; its contribution is additive and contextual.

## 5 Mendelian randomization: what the evidence establishes and what it does not

Ference et al. (4) used genetic instruments from CETP and HMGCR variants in 102,837 participants with 13,821 major cardiovascular events, validated in 189,539 participants from CARDIoGRAMplusC4D. The CETP + HMGCR combination produces equivalent LDL-C reduction with attenuated ApoB reduction, a genetically induced discordance that allows the two variables to be causally disentangled.

The discordant genetic score, same LDL-C reduction, less ApoB reduction, produced no cardiovascular benefit (OR 0.985; 95% CI 0.955–1.015; *p* non-significant). Concordant ApoB reduction produced consistent protection across all four variant classes: CETP alone (OR 0.781 per 10 mg/dL ApoB reduction), HMGCR (OR 0.794), PCSK9 (OR 0.787), NPC1L1 (OR 0.786), with no statistically significant difference between classes (*p* = 0.79).

What this MR establishes: ApoB particle count is causally related to cardiovascular risk, and reducing cholesterol content without reducing particle count produces no cardiovascular benefit. What it does not establish: whether, among individuals with equal ApoB concentration, the RT of each particle is an additional independent risk determinant. RT requires direct stable-isotope measurement and is not available as a genetic instrument in standard MR studies.

One MR finding directly supports the RT framework: complete genetic CETP deficiency (Ikewaki et al. (53) produces both a 44%–92% FCR increase and a 55%–81% ApoB production reduction, reducing the product [ApoB] x (1/FCR) by two concurrent routes. Pharmacological CETP inhibitors without FCR effect act only on cholesterol content. This asymmetry explains why CETP genetic variants produce greater risk reduction per unit LDL-C than pharmacological inhibitors in Ference's analysis: the genetic variant exploits the kinetic route; the drug does not.

## 6 Relationship to competing frameworks

### 6.1 Response-to-retention model (williams and tabas)

Williams and Tabas formulated that the proximal determinant of atherogenesis is the retention time of the ApoB particle in the subendothelial proteoglycan matrix, not in plasma (54). This model has solid experimental support: murine studies demonstrated that LDL particles engineered not to interact with proteoglycans are not atherogenic regardless of plasma concentration.

The relationship between plasma RT and subendothelial retention is sequential complementarity, not competition. Plasma RT determines which particle reaches the subendothelium and in what oxidative state; the retention model determines what happens once it arrives. Elevated plasma RT amplifies retention by two routes: first, generating L5, which has four-fold higher aortic proteoglycan binding affinity than native LDL (58); oxidized LDL degrades the glycocalyx gate (Group 3 mechanism), increasing transcapillary access. The two models are best understood as serial steps in a single continuous process.

### 6.2 Inflammatory model (ridker-CANTOS)

CANTOS demonstrated that canakinumab anti-interleukin-1 beta [IL-1beta] reduces recurrent cardiovascular events by 15% relative risk reduction in post-MI patients with elevated hsCRP, without lipid modification (55). The RT framework is not contradicted by CANTOS: IL-1beta activates downstream cascades including IL-6 and TNF-alpha, which elevate kmod and generate the Group 2 kinetic dead zone. Under the RT framework, canakinumab reduces events by reducing kmod, restoring FCR_cin, shortening RT, and reducing L5 generation. Whether benefit is proportional to baseline kmod markers (hsCRP, oxLDL, L5 fraction) is a sub-analysis not yet published and would constitute genuine evidence for or against this interpretation.

### 6.3 Relationship to the CKM syndrome framework

The cardiovascular-kidney-metabolic (CKM) syndrome framework (Ndumele et al., (59)) integrates obesity, metabolic syndrome, chronic kidney disease, and cardiovascular disease as a continuous pathophysiological spectrum. The RT framework is not in competition with CKM. It provides a mechanistic substrate for the lipid-atherogenic component of CKM. The f(UACR, glycocalyx) term formalizes the kidney-vascular axis at the level of endothelial glycocalyx integrity; the kmod term captures the metabolic environment component that CKM stages 2–3 describe phenotypically. Three distinctions are worth making explicit:

First, CKM is a clinical staging system built on observable phenotypes. It does not generate falsifiable mechanistic predictions at the particle level. It cannot predict what happens to the L5 fraction in a PCSK9 LOF carrier, or why FCR increases with weight loss in metabolic syndrome but not in hemodialysis. The RT equation generates those predictions, and they are falsifiable with stable-isotope kinetics and anion-exchange chromatography. CKM describes the terrain; the RT framework proposes a mechanism that could be proven wrong.

Second, CKM retains the individual patient as the primary unit of analysis. The RT framework deliberately shifts the focus to the metabolic environment as the determinant of kmod, opening the analytical frame to variables that operate above the individual level, including chronic exposure to environmental oxidative stressors and inflammatory burden from social determinants of health. The kmod is not a property of the patient; it is a property of the patient-environment interaction.

Third, CKM does not resolve the paradoxes that motivated this hypothesis. Two patients at CKM stage 3 with identical clinical phenotypes can have radically different cardiovascular outcomes depending on which kmod pathway is dominant (carbamylation in uremia versus NADPH oxidase activation in autoimmunity versus Maillard glycation in established T2DM). That mechanistic heterogeneity is invisible to CKM staging but is the central explanatory target of the RT framework.

## 7 Falsifiable predictions and research agenda

The predictions below distinguish the RT framework from the ApoB-years model in ways testable with currently available technology. Each prediction is stated with the specific finding that would refute it.

### 7.1 P1

L5 fraction inversely proportional to PCSK9 activity. Prediction: L5 fraction (anion-exchange chromatography) is significantly lower in PCSK9 LOF carriers than in GOF carriers and controls, adjusted for total LDL-C. Refutation: no difference in L5 fraction between LOF and GOF at equivalent LDL-C.

### 7.2 P2

FCR-mechanism variants produce greater [ApoB x RT] reduction than production-mechanism variants at equivalent final ApoB. Prediction: combined stable-isotope kinetic study plus simultaneous L1-L5 subfraction profiling in Group 1a (FH heterozygous), Group 2 (metabolic syndrome, matched ApoB), and controls will show FCR differences with equivalent ApoB but different L5 profiles. Refutation: no difference in FCR or L5 profile between groups at equivalent ApoB.

### 7.3 P3

iPCSK9 benefit is a function of RT reduction, not prior cumulative ApoB exposure. Prediction: sub-analysis of FOURIER and ODYSSEY OUTCOMES stratified by treatment duration and FCR will show that patients initiating iPCSK9 at age 60 who achieve FCR equivalent to LOF carriers obtain the same reduction in L5 fraction as genetic LOF carriers of the same age. Refutation: benefit per unit FCR increase declines significantly with age at treatment initiation.

### 7.4 P4

Elevated L5 with normal or low ApoB in high-volume male endurance athletes with elevated CAC. Prediction: male athletes with CAC score >100 have significantly higher L5 fraction than athletes with CAC=0 and sedentary controls with equivalent ApoB. Refutation: no L5 elevation in high-CAC athletes compared to low-CAC athletes at equivalent ApoB.

### 7.5 P5

UACR explains residual cardiovascular risk within equivalent ApoB strata. Prediction: in a prospective cohort with measured ApoB and UACR, UACR adds significant independent predictive value for cardiovascular events within ApoB quintiles. Refutation: UACR adds no predictive value after adjustment for ApoB.

## 8 Limitations

Table 3 summarizes epistemic confidence by component of the framework. The central limitation is structural: the RT model has not been validated in a prospective study with RT measured directly. Every causal claim about RT's role in atherogenesis is currently inferential, derived from *in vitro* and *ex vivo* kinetic studies (conditions may not replicate *in vivo* biology), stable-isotope kinetic studies in small human samples (Pietzsch et al.: *n* = 6 and *n* = 7), and *post-hoc* narrative consistency between the model and pre-existing clinical observations. The epistemic distinction between generating coherent *post-hoc* explanations and prospectively predicting data is real and must not be elided.

Circular reasoning risk. The natural experiments of Section 3 were used to construct the biological narrative, and that narrative is then used to explain those same situations. This is the standard architecture of hypothesis papers. The risk becomes an actual problem when narrative consistency is presented as predictive validation. The explanatory claims of Section 3 should be treated as *post-hoc* coherence arguments evaluated by their internal logic and mechanistic specificity, not as independent validation.

The kinetic dead zone mechanism, central to Group 2, rests primarily on *in vitro* evidence. Glycocalyx evidence is predominantly from animal models. kmod lacks a prospectively validated clinical proxy: hsCRP, oxLDL, and PON1 are partial proxies. L5 is elevated in conditions as diverse as FH, hypertriglyceridemia, diabetes, hemodialysis, and post-aerobic exercise, complicating its use as a specific kmod biomarker and requiring context stratification before clinical interpretation.

The RT -> L5 -> RT positive feedback loop has not been measured as an integrated system in humans. Individual components are independently demonstrated; the circuit as a whole has not been characterized in a single *in vivo* human experiment. L5 has not been directly measured in PCSK9 variant carriers, the most directly falsifiable prediction of the model and the most notable gap in existing data.

## 9 Conclusions

Ference et al. established that cardiovascular risk follows ApoB particle count, not cholesterol content. The 2026 ACC/AHA guideline formalized that advance. The step this paper proposes is to recognize that equal ApoB particle counts generate radically different cardiovascular risk depending on how long each particle remains in circulation and in what oxidative conditions it does so.

Seven documented clinical paradoxes, from randomized trials with negative results to reproducible epidemiological findings, constitute natural experiments where the particle-count model fails and where the RT x kmod x f(glycocalyx) framework generates mechanistically coherent, internally consistent predictions (Table 2). The resulting functional classification generates qualitatively distinct therapeutic implications: the rate-limiting step is the receptor in Group 1a, the ligand in Group 2, and the endothelial gate in Group 3. Treating these groups with the same LDL-C reduction strategy is a category error with quantifiable consequences.

**Table 2:** Explanatory capacity of three models across selected clinical paradoxes.

Clinical situation	LDL-C model	ApoB-years model	RT framework (mechanism)
Hemodialysis: 10–20x CV risk with normal ApoB; statin failure (4D, AURORA)	No	No	Uremic kmod -> kinetic dead zone -> FCR_cin reduced; statins cannot repair chemically modified ligand (40)
Evacetrapib/dalcetrapib: LDL-C −30–40%, no CV benefit	No	Partial	RT unchanged; cholesterol emptied without withdrawing particle; L5 sequence continues
Anacetrapib: CV benefit proportional to ApoB reduction	No	Yes	FCR +23% by stable isotopes (Millar et al. (7) -> RT reduced; mechanism confirmed
PCSK9 LOF: 88% CHD reduction at LDL-C 100 mg/dL, 3x what dose-response predicts	No	Partial	RT ∼1.4 days (Cariou et al. (10) -> latency phase, no L5; plus reduced LOX-1 upregulation (23): dual mechanism
SLE/RA: accelerated atherosclerosis with normal or low LDL-C; RA lipid paradox	No	No	Chronic inflammatory kmod (TNF-alpha, IL-6) elevates L5 independently of LDL-C; L5 x3.4 in SLE (44); OR 4.94 for atherosclerosis in RA (45)
Antioxidant trial failure (HOPE, CARET, SELECT)	N/A	N/A	kmod is episodic; stationary coverage cannot neutralize peaks; high-dose vitamin E inhibits GST-pi (48) and attenuates NRF2 adaptive response (47)
Male endurance athletes: elevated CAC despite lower ApoB	No	Contradicted	Episodic Nox2 kmod + glycocalyx degradation; estradiol protects glycocalyx and ApoB conformation (49); explains female exemption

**Table 3:** Epistemic confidence by component of the RT framework.

Component	Confidence	Key basis and limiting factor
FCR as determinant of RT	High	Stable-isotope kinetics in humans; multiple independent groups since Bilheimer et al. 1979 (60)
ApoB count as causal variable (MR)	High	Ference et al. MR, 102,837 + 189,539 participants (4); UK Biobank replication Morze et al. (6)
Oxidative modification sequence and L5 generation	Moderate	*In vitro* well-characterized; Pietzsch et al. *in vivo* correlation (*n* = 6/7, CI wide) (21); L5 clinical data consistent but small samples
Kinetic dead zone in uremia (Group 2 core)	Moderate	*In vitro* mechanism; Ikewaki et al. direct FCR measurement in dialysis patients (40); L5 elevation in four independent studies; no randomized test of mechanism reversal
kmod x f(UACR) as integrated atherogenic load predictor	Low-moderate	Mechanistically plausible; no prospective validation of equation as clinical predictive tool; no validated kmod proxy
Group 3: glycocalyx gate degradation	Low	Predominantly animal data; UACR as glycocalyx proxy not validated prospectively for this mechanism; presented as second-level hypothesis
RT -> L5 -> RT feedback loop as integrated system	Low	Individual components demonstrated; complete circuit not measured as system in humans

This paper does not demonstrate that the RT model is correct. It demonstrates that the paradoxes of the classical model are systematic and point in the same mechanistic direction, that a hypothesis consistent with available kinetic evidence can organize them in a single framework, and that the framework generates specific falsifiable predictions testable with currently available technology. The five predictions in Section 7 define both the research agenda and the conditions under which this framework should be revised or abandoned if the data do not support it.

## References

[1] Baigent C Blackwell L Emberson J. Efficacy and safety of more intensive lowering of LDL cholesterol: a meta-analysis of data from 170,000 participants in 26 randomised trials. Lancet. (2010) 376(9753):1670–81. 10.1016/S0140-6736(10)61350-5 21067804 PMC2988224

[2] Collins R Armitage J Parish S. MRC/BHF heart protection study of cholesterol lowering with simvastatin in 20,536 high-risk individuals: a randomised placebo-controlled trial. Lancet. (2002) 360(9326):7–22. 10.1016/S0140-6736(02)09327-3 12114036

[3] Ference BA Yoo W Alesh I Mahajan N Mirowska KK Mewada A. Effect of long-term exposure to lower low-density lipoprotein cholesterol beginning early in life on the risk of coronary heart disease. J Am Coll Cardiol. (2012) 60(25):2361–9. 10.1016/j.jacc.2012.09.017 23083789

[4] Ference BA Kastelein JJP Ginsberg HN Chapman MJ Nicholls SJ Ray KK. Association of genetic variants related to CETP inhibitors and statins with lipoprotein levels and cardiovascular risk. JAMA. (2017) 318(10):947–56. 10.1001/jama.2017.11467 28846118 PMC5710502

[5] Blumenthal RS Morris PB Gaudino M Johnson HM Anderson TS Bittner VA. 2026 ACC/AHA/AACVPR/ABC/ACPM/ADA/AGS/APhA/ASPC/NLA/PCNA guideline on the management of dyslipidemia. J Am Coll Cardiol. (2026) 87(19):2624–757. 10.1016/j.jacc.2025.11.016 41824590

[6] Morze J Melloni GEM Wittenbecher C Ala-Korpela M Rynkiewicz A Guasch-Ferré M. ApoB-containing lipoproteins: count, type, size, and risk of coronary artery disease. Eur Heart J. (2025) 46(27):2691–701. 10.1093/eurheartj/ehaf207 40289348

[7] Millar JS Reyes-Soffer G Jumes P Dunbar RL Degoma EM Baer AL. Anacetrapib lowers LDL by increasing ApoB clearance in mildly hypercholesterolemic subjects. J Clin Invest. (2015) 125(6):2510–22. 10.1172/JCI80025 25961461 PMC4497759

[8] Bowman L Hopewell JC Chen F. Effects of anacetrapib in patients with atherosclerotic vascular disease. N Engl J Med. (2017) 377(13):1217–27. 10.1056/NEJMoa1706444 28847206

[9] Cohen JC Boerwinkle E Mosley TH Jr Hobbs HH. Sequence variations in PCSK9, low LDL, and protection against coronary heart disease. N Engl J Med. 2006 354(12):1264–72. 10.1056/NEJMoa054013 16554528

[10] Cariou B Ouguerram K Zaïr Y Guerois R Langhi C Kourimate S. PCSK9 Dominant negative mutant results in increased LDL catabolic rate and familial hypobetalipoproteinemia. Arterioscler Thromb Vasc Biol. (2009) 29(12):2191–7. 10.1161/ATVBAHA.109.194191 19762784

[11] Daïen CI Duny Y Barnetche T Daurès JP Combe B Morel J. Effect of TNF inhibitors on lipid profile in rheumatoid arthritis: a systematic review with meta-analysis. Ann Rheum Dis. (2012) 71(6):862–8. 10.1136/annrheumdis-2011-201148 22267329

[12] Sniderman AD Thanassoulis G Glavinovic T Navar AM Pencina M Catapano A. Apolipoprotein B particles and cardiovascular disease: a narrative review. JAMA Cardiol. (2019) 4(12):1287–95. 10.1001/jamacardio.2019.3780 31642874 PMC7369156

[13] Elovson J Chatterton JE Bell GT Schumaker VN Reuben MA Puppione DL Reeve JR Jr Young NL. Plasma very low density lipoproteins contain a single molecule of apolipoprotein B. J Lipid Res. 1988 29(11):1461–73 10.1016/S0022-2275(20)38425-X 3241122

[14] Cheng MJ Kumar R Sridhar S Webster TJ Ebong EE. Endothelial glycocalyx conditions influence nanoparticle uptake for passive targeting. Int J Nanomedicine. (2016) 11:3305–15. 10.2147/IJN.S106299 27499624 PMC4959595

[15] Cheng MJ Mitra R Okorafor CC Nersesyan AA Harding IC Bal NN. Targeted intravenous nanoparticle delivery: role of flow and endothelial glycocalyx integrity. Ann Biomed Eng. (2020) 48(7):1941–54. 10.1007/s10439-020-02474-4 32072383 PMC8025840

[16] Kang H Yan G Lin X Tian Y Yin J Liu J. The substructure of the endothelial glycocalyx in rat aorta and its interaction with low-density lipoproteins. Am J Pathol. (2025) 195(10):1936–58. 10.1016/j.ajpath.2025.06.005 40645580 PMC12597546

[17] Nicoll A Duffield R Lewis B. Flux of plasma lipoproteins into human arterial intima: comparison of grossly normal and atheromatous intima. Atherosclerosis. (1981) 39(2):229–42. 10.1016/0021-9150(81)90073-3 7248000

[18] Jorgensen AB Frikke-Schmidt R Nordestgaard BG Tybjaerg-Hansen A. Loss-of-function mutations in APOC3 and risk of ischemic vascular disease. N Engl J Med. (2014) 371(1):32–41. 10.1056/NEJMoa1308027 24941082

[19] Graham MJ Lee RG Brandt TA Tai LJ Fu W Peralta R. Cardiovascular and metabolic effects of ANGPTL3 antisense oligonucleotides. N Engl J Med. (2017) 377(3):222–32. 10.1056/NEJMoa1701329 28538111

[20] Tertov VV Kaplun VV Sobenin IA Orekhov AN. Low-density lipoprotein modification occurring in human plasma: possible mechanism of *in vivo* lipoprotein desialylation as a primary step of atherogenic modification. Atherosclerosis. (1998) 138(1):183–95. 10.1016/s0021-9150(98)00023-9 9678784

[21] Pietzsch J Lattke P Julius U. Oxidation of apolipoprotein B-100 in circulating LDL is related to LDL residence time: *in vivo* insights from stable isotope studies. Arterioscler Thromb Vasc Biol. (2000) 20(10):e63–7. 10.1161/01.atv.20.10.e63 11031225

[22] Hu B Li D Sawamura T Mehta JL. Oxidized low-density lipoprotein through LOX-1 modulates LDL-receptor expression in human coronary artery endothelial cells. Biochem Biophys Res Commun. (2003) 307(4):1008–12. 10.1016/s0006-291x(03)01295-6 12878212

[23] Ding Z Pothineni NVK Goel A Lüscher TF Mehta JL. PCSK9 And inflammation: role of shear stress, pro-inflammatory cytokines, and LOX-1. Cardiovasc Res. (2020) 116(5):908–15. 10.1093/cvr/cvz313 31746997

[24] Rivas-Urbina A Rull A Ordonez-Llanos J Sanchez-Quesada JL. Electronegative LDL: an active player in atherogenesis or a by-product of atherosclerosis? Curr Med Chem. (2019) 26(9):1665–79. 10.2174/0929867325666180330093953 29600751

[25] Kang H Yang J Zhang W Lu J Ma X Sun A. Effect of endothelial glycocalyx on water and LDL transport through the rat abdominal aorta. Am J Physiol Heart Circ Physiol. (2021) 320(4):H1724–37. 10.1152/ajpheart.00861.2020 33710913

[26] Constantinescu AA Vink H Spaan JA. Elevated capillary tube hematocrit reflects degradation of endothelial cell glycocalyx by oxidized LDL. Am J Physiol Heart Circ Physiol. (2001) 280(3):H1051–7. 10.1152/ajpheart.2001.280.3.H1051 11179046

[27] Rabelink TJ De Zeeuw D. The glycocalyx - linking albuminuria with renal and cardiovascular disease. Nat Rev Nephrol. (2015) 11(11):667–76. 10.1038/nrneph.2015.162 26460356

[28] Salmon AH Satchell SC. Endothelial glycocalyx dysfunction in disease: albuminuria and increased microvascular permeability. J Pathol. (2012) 226(4):562–74. 10.1002/path.3964 22102407

[29] Wanner C Krane V März W Olschewski M Mann JFE Ruf G. Atorvastatin in patients with type 2 diabetes mellitus undergoing hemodialysis. N Engl J Med. (2005) 353(3):238–48. 10.1056/NEJMoa043545 16034009

[30] Fellström BC Jardine AG Schmieder RE Holdaas H Bannister K Beutler J. Rosuvastatin and cardiovascular events in patients undergoing hemodialysis. N Engl J Med. (2009) 360(14):1395–407. 10.1056/NEJMoa0810177 19332456

[31] Apostolov EO Shah SV Ok E Basnakian AG. Quantification of carbamylated LDL in human sera by a new sandwich ELISA. Clin Chem. (2005) 51(4):719–28. 10.1373/clinchem.2004.044032 15684275

[32] Apostolov EO Ray D Savenka AV Shah SV Basnakian AG. Chronic uremia stimulates LDL carbamylation and atherosclerosis. J Am Soc Nephrol. (2010) 21(11):1852–7. 10.1681/ASN.2010040365 20947625 PMC3014000

[33] Wang Z Nicholls SJ Rodriguez ER Kummu O Hörkkö S Barnard J. Protein carbamylation links inflammation, smoking, uremia and atherogenesis. Nat Med. (2007) 13(10):1176–84. 10.1038/nm1637 17828273

[34] Kobayashi M Watanabe K Suzuki T Dohmae N Fujiyoshi M Uchida M. Analysis of the acrolein-modified sites of apolipoprotein B-100 in LDL. Biochim Biophys Acta Mol Cell Biol Lipids. (2020) 1866(1):158809. 10.1016/j.bbalip.2020.158809 32919080

[35] Watanabe K Nakazato Y Saiki R Igarashi K Kitada M Ishii I. Acrolein-conjugated low-density lipoprotein induces macrophage foam cell formation. Atherosclerosis. (2012) 227(1):51–7. 10.1016/j.atherosclerosis.2012.12.020 23305793

[36] Chang C-T Wang G-J Kuo C-C Hsieh J-Y Lee A-S Chang C-M. Electronegative low-density lipoprotein increases coronary artery disease risk in uremia patients on maintenance hemodialysis. Medicine (Baltimore). (2016) 95(2):e2265. 10.1097/MD.0000000000002265 26765403 PMC4718229

[37] Lobo JC Mafra D Farage NE Faulin TES Abdalla DSP De Nóbrega ACL. Increased electronegative LDL and decreased antibodies against electronegative LDL levels correlate with inflammatory markers and adhesion molecules in hemodialysed patients. Clin Chim Acta. (2011) 412(19-20):1788–92. 10.1016/j.cca.2011.05.034 21676364

[38] Hirowatari Y Homma Y Yoshizawa J Homma K. Increase of electronegative-LDL-fraction ratio and IDL-cholesterol in chronic kidney disease patients with hemodialysis treatment. Lipids Health Dis. (2012) 11:111. 10.1186/1476-511X-11-111 22962943 PMC3539864

[39] Chang KC Lee AS Chen WY Lin YN Hsu JF Chan HC. Increased LDL electronegativity in chronic kidney disease disrupts calcium homeostasis resulting in cardiac dysfunction. J Mol Cell Cardiol. (2015) 84:36–44. 10.1016/j.yjmcc.2015.03.016 25871829

[40] Ikewaki K Schaefer JR Frischmann ME Okubo K Hosoya T Mochizuki S. Delayed *in vivo* catabolism of intermediate-density lipoprotein and low-density lipoprotein in hemodialysis patients as potential cause of premature atherosclerosis. Arterioscler Thromb Vasc Biol. (2005) 25(12):2615–22. 10.1161/01.ATV.0000188555.60475.c2 16195474

[41] Lincoff AM Nicholls SJ Riesmeyer JS Barter PJ Brewer HB Fox KAA. Evacetrapib and cardiovascular outcomes in high-risk vascular disease. N Engl J Med. (2017) 376(20):1933–42. 10.1056/NEJMoa1609581 28514624

[42] Manzi S Meilahn EN Rairie JE Conte CG Medsger TA Jansen-McWilliams L. Age-specific incidence rates of myocardial infarction and angina in women with systemic lupus erythematosus: comparison with the framingham study. Am J Epidemiol. (1997) 145(5):408–15. 10.1093/oxfordjournals.aje.a009122 9048514

[43] Giles JT Wasko MCM Chung CP Szklo M Blumenthal RS Kao A. Exploring the lipid paradox theory in rheumatoid arthritis: associations of low circulating low-density lipoprotein concentration with subclinical coronary atherosclerosis. Arthritis Rheumatol. (2019) 71(9):1426–36. 10.1002/art.40889 30883031 PMC6716986

[44] Chan HC Liang CJ Lee HC Su H Lee AS. Role of low-density lipoprotein in early vascular aging associated with systemic lupus erythematosus. Arthritis Rheumatol. (2020) 72(6):972–84. 10.1002/art.41213 31994323

[45] Chang CY Chen CH Chen YM Hsieh TY Li JP Shen MY. Association between negatively charged low-density lipoprotein L5 and subclinical atherosclerosis in rheumatoid arthritis patients. J Clin Med. (2019) 8(2):177. 10.3390/jcm8020177 30717448 PMC6406888

[46] Forman HJ Davies KJ Ursini F. How do nutritional antioxidants really work: nucleophilic tone and para-hormesis versus free radical scavenging *in vivo*. Free Radic Biol Med. (2013) 66:24–35. 10.1016/j.freeradbiomed.2013.05.045 23747930 PMC3852196

[47] Tebay LE Robertson H Durant ST Vitale SR Penning TM Dinkova-Kostova AT. Mechanisms of activation of the transcription factor Nrf2 by redox stressors, nutrient cues, and energy status and the pathways through which it attenuates degenerative disease. Free Radic Biol Med. (2015) 88(Pt B):108–46. 10.1016/j.freeradbiomed.2015.06.021 26122708 PMC4659505

[48] Van Haaften RI Evelo CT Haenen GR Bast A. Alpha-tocopherol inhibits human glutathione S-transferase pi. Biochem Biophys Res Commun. (2001) 280(2):631–3. 10.1006/bbrc.2000.4174 11162567

[49] Brunelli R Balogh G Costa G De Spirito M Greco G Mei G. Estradiol binding prevents apoB-100 misfolding in electronegative LDL(-). Biochemistry. (2010) 49(34):7297–302. 10.1021/bi100715f 20669963

[50] Tremblay AJ Lamarche B Ruel IL Hogue J-C Bergeron J Gagné C. Increased production of VLDL apoB-100 in subjects with familial hypercholesterolemia carrying the same null LDLR mutation. J Lipid Res. (2004) 45(5):866–72. 10.1194/jlr.M300448-JLR200 14967814

[51] Ng TW Watts GF Barrett PH Rye KA Chan DC. Effect of weight loss on LDL and HDL kinetics in the metabolic syndrome. Diabetes Care. (2007) 30(11):2945–50. 10.2337/dc07-0768 17686833

[52] Richard C Couture P Ooi EMM Tremblay AJ Desroches S Charest A. Effect of Mediterranean diet with and without weight loss on apolipoprotein B100 metabolism in men with metabolic syndrome. Arterioscler Thromb Vasc Biol. (2013) 34(2):433–8. 10.1161/ATVBAHA.113.302185 24265415

[53] Ikewaki K Nishiwaki M Sakamoto T Ishikawa T Fairwell T Zech LA. Increased catabolic rate of low density lipoproteins in humans with cholesteryl ester transfer protein deficiency. J Clin Invest. (1995) 96(3):1573–81. 10.1172/JCI118196 7657828 PMC185783

[54] Williams KJ Tabas I. The response-to-retention hypothesis of early atherogenesis. Arterioscler Thromb Vasc Biol. (1995) 15(5):551–61. 10.1161/01.atv.15.5.551 7749869 PMC2924812

[55] Ridker PM Everett BM Thuren T MacFadyen JG Chang WH Ballantyne C. Antiinflammatory therapy with canakinumab for atherosclerotic disease. N Engl J Med. (2017) 377(12):1119–31. 10.1056/NEJMoa1707914 28845751

[56] Parhofer KG Hugh P Barrett R Bier DM Schonfeld G. Determination of kinetic parameters of apolipoprotein B metabolism using amino acids labeled with stable isotopes. J Lipid Res. (1991) 32(8):1311–23. 10.1016/S0022-2275(20)41961-3 1770313

[57] Welty FK Lichtenstein AH Barrett PHR Dolnikowski GG Schaefer EJ. Human apolipoprotein (Apo) B-48 and apoB-100 kinetics with stable isotopes.. Arterioscler Thromb Vasc Biol. (1999) 19(12):2966–74. 10.1161/01.ATV.19.12.2966 10591677

[58] Bancells C Benítez S Jauhiainen M Ordóñez-Llanos J Kovanen PT Villegas S. High binding affinity of electronegative LDL to human aortic proteoglycans depends on its aggregation level. J Lipid Res. (2008) 50(3):446–55. 10.1194/jlr.M800318-JLR200 18952981

[59] Ndumele CE Rangaswami J Chow SL Neeland IJ Tuttle KR Khan SS. Cardiovascular-kidney-metabolic health: a presidential advisory from the American Heart Association. Circulation. (2023) 148(20):1606–35. 10.1161/CIR.0000000000001184 37807924

[60] Bilheimer DW Stone NJ Grundy SM. Metabolic studies in familial hypercholesterolemia: evidence for a gene-dosage effect *in vivo*. J Clin Invest. (1979) 64(2):524–33. 10.1172/JCI109490 222811 PMC372147

